# Physical Function in People with Disabilities and Social Determinants: A Growth Mixture Modeling Study

**DOI:** 10.1093/geroni/igaf122.3363

**Published:** 2025-12-31

**Authors:** Seeun Park, Ivan Molton

**Affiliations:** University of Washington, Seattle, Washington, United States; University of Washington, Seattle, Washington, United States

## Abstract

Preservation of physical functioning is crucial for successful aging among people with disabilities (PWD). While physical function is influenced by social determinants of health (SDOH) in the general population, its relationship with PWD remains unclear. This study aimed to identify trajectories of physical functioning and examine the role of SDOH among PWD. This study utilized two years of longitudinal data from the Rehabilitation Research and Training Center on Healthy Aging and Physical Disability at the University of Washington. Participants were community-dwelling adults with disabilities, including those with spinal cord injury, multiple sclerosis, muscular dystrophy, or post-polio syndrome. Physical function was assessed using the PROMIS Physical Function measure. SDOH factors included health service availability, provider relationships, financial and physical barriers to healthcare, disability benefits, transportation accessibility, community accessibility, employment support, and caregiving availability. Growth Mixture Modeling was used to identify distinct physical function trajectory classes, and multinomial logistic regression examined the associations between SDOH and trajectory membership. A total of 1,573 PWD were included. Four distinct trajectory classes were identified: High Stable, High Decline, Moderate Decline, and Low Function. Those in the Low Function class were more likely to experience disadvantaged SDOH, including lower income, greater physical barriers to healthcare, reduced community accessibility, and lack of disability benefits. Similar trends were observed in the High Decline and Moderate Decline classes. Findings highlight the significant role of SDOH in shaping physical function trajectories among PWD, emphasizing the need to improve healthcare accessibility by addressing financial and physical barriers and fostering social inclusion.

